# Adaptive auditory brightness perception

**DOI:** 10.1038/s41598-021-00707-7

**Published:** 2021-11-02

**Authors:** Kai Siedenburg, Feline Malin Barg, Henning Schepker

**Affiliations:** 1 Department of Medical Physics and Acoustics, Carl von Ossietzky University of Oldenburg, Oldenburg, Germany; 2 Starkey Hearing, Eden Prairie, MN USA

**Keywords:** Auditory system, Psychology, Human behaviour

## Abstract

Perception adapts to the properties of prior stimulation, as illustrated by phenomena such as visual color constancy or speech context effects. In the auditory domain, only little is known about adaptive processes when it comes to the attribute of auditory brightness. Here, we report an experiment that tests whether listeners adapt to spectral colorations imposed on naturalistic music and speech excerpts. Our results indicate consistent contrastive adaptation of auditory brightness judgments on a trial-by-trial basis. The pattern of results suggests that these effects tend to grow with an increase in the duration of the adaptor context but level off after around 8 trials of 2 s duration. A simple model of the response criterion yields a correlation of r = .97 with the measured data and corroborates the notion that brightness perception adapts on timescales that fall in the range of auditory short-term memory. Effects turn out to be similar for spectral filtering based on linear spectral filter slopes and filtering based on a measured transfer function from a commercially available hearing device. Overall, our findings demonstrate the adaptivity of auditory brightness perception under realistic acoustical conditions.

## Introduction

Whether sounds are perceived as *low* or *high*, *bright* or *dull*, *shrill* or *noisy*, depends to a large part on the sound spectrum. In many everyday-life acoustical context, however, the spectral properties of one and the same source sound may differ considerably at the eardrum of the listener due to coloration effects from specific room-acoustics, reverberation, sound recording, sound reproduction, or processing in hearing devices. An important auditory attribute that can be directly affected by spectral coloration is auditory brightness. The latter depends on the balance of low to high frequency energy in a sound and constitutes a central component of a sound’s timbre^1–4^. Even though context effects have been studied extensively in auditory perception^5,6^, it still remains unclear as to whether (and on which timescale) brightness perception adapts to properties of acoustic contexts with distinct colorations. Understanding the role of context-based adaptation in brightness perception thus is an important step for developing a comprehensive understanding of auditory perception under realistic conditions.

Context effects in auditory perception have long been studied with categorization tasks that focus on sound identity (e.g., /ga/ vs. /da/, violin vs. flute). Early studies showed that spectral properties of a preceding speech context affect the categorization of vowel targets^7^. Studies by Holt^5^ showed that the categorization of sounds along a /ga/–/da/ continuum, for instance, is affected by the statistical distribution of non-adjacent, non-speech context sounds, suggesting a general auditory mechanism of spectral contrast enhancement. Schweinberger and colleagues^8^ demonstrated voice adaptation in speaker gender categorization and later indicated that spectral envelope cues more strongly contribute to the voice adaptation effect compared to fundamental frequency (F0) cues^9^. Auditory adaptation has also been thoroughly documented for musical instrument categorization^10–13^. In these studies, listeners categorized instrument sounds along a continuum between, say, a French horn and a saxophone, each with the same F0 but different spectral envelopes. Recently, it was observed that adaptation effects also emerge for unprocessed musical stimuli^14^. Studies have suggested both central and peripheral origins of these spectral contrast effects^15,16^.

Regarding more elementary sound attributes, it has long been known that pitch discrimination declines when tested with frequency-roving test tones^17,18^. Raviv et al.^19^ specifically reported a *contraction bias* in pitch discrimination, where listeners tended to indicate that the 2nd tone of a pair was higher in pitch when both tones were generally relatively high, and reversely, listeners indicated that the 1st tone was higher when both tones were relatively low. Considering frequency shift judgements, Chambers et al.^20^ demonstrated long-lasting effects of a prior context, which was later shown to extend to brightness shifts^21^. Representations of sound level have similarly been shown to be subject to adaptation over time^22^. Together these findings suggest that even the computation of elementary auditory attributes features aspects of recalibration according to the statistics of sensory signals encountered in the recent past. The goal of the present study was to test this hypothesis for the attribute of auditory brightness.

Several methods by which spectral contrast effects in auditory categorization have been measured, whether with speech or musical sounds, remain limited. Participants are usually exposed to test sounds generated by morphing two distinct sounds and hence only encounter subtle variations of the same two sounds throughout a whole experimental session^8,11–13^. Furthermore, the typical adaptation paradigm separates the exposure and test phase by presenting an adaptor stimulus (i.e., an auditory context often consisting of multiple presentations of one stimulus from an end or middle point of the continua), followed by the test stimulus. Thus, precious temporal information regarding the adaptation process is lost, because it is unclear when and with which magnitude potential effects set in. In fact, studies on adaptation to frequency modulation showed that adaptation effects may emerge after a single trial of 100-ms duration^23^. Similarly, in a study on sound texture discrimination, effects of temporal integration were observed within a single trial^24^.

Here, we tested the degree to which auditory brightness of naturalistic music and speech excerpts is subject to adaptation over time. A series of four experiments demonstrated small but consistent adaptation of brightness judgments on a trial-by-trial basis. Brightness was varied by altering the spectral balance of the stimuli using a filterbank, as well as by morphing between the acoustical transfer function of the open ears of a dummy head and the ear of the same dummy head aided with commercially available hearing devices. Using an experimental task that does not require any repetition of stimuli within an experimental session, we observed a distinct temporal signature of brightness adaptation that was corroborated by signal-based model of the the decision-criterion.

## Results

**Figure 1:**
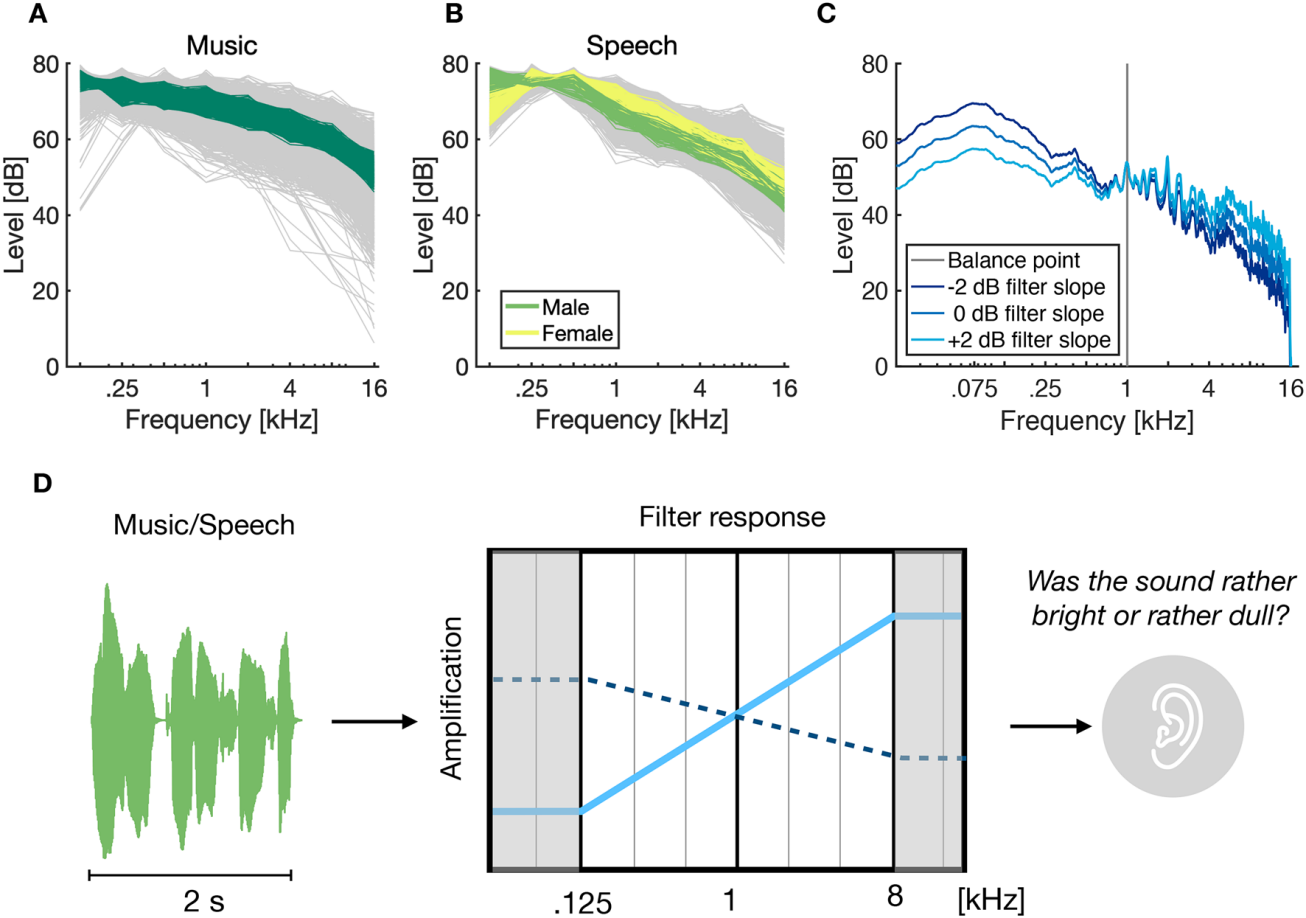
**(A)** Distribution of spectral energy levels of broadband music excerpts measured between 0.125 and 16 kHz (one-octave-spacing) for all music excerpts in the database (gray lines) and the used test excerpts (dark green). **(B)** Distribution of spectral energy levels for broadband speech excerpts in the database (gray lines) and the test excerpts of female speakers (yellow lines) and male speakers (green lines) between 0.125 and 16 kHz. **(C)** Exemplary magnitude responses of speech samples filtered with different filter slopes. **(D)** Illustration of the experimental task in all four experiments: on every trial, participants heard a filtered excerpt of music or speech and judged whether the excerpt sounded rather *bright* or *dull* according to their internal reference. The solid light blue line as filter response represents a high-pass characteristic with positive spectral filter slope; the dashed dark blue line represents a low-pass characteristic with negative spectral filter slope. Filter weights remained constant below 125 Hz and above 16 kHz.

We tested adaptation of auditory brightness perception by asking listeners to indicate whether stimuli sounded *rather bright* or *rather dull*. Short excerpts from pieces of popular music or speech utterances were presented. To modify brightness, stimuli were filtered to change the spectral slope of sounds between 0.125 and 8 kHz. See Fig. 1A–C for the spectral shape of the stimuli and Fig. 1D for a schematic of the experimental task. The different filtering conditions were presented in a random order. Every individual stimulus was presented only once per experimental session, hence, learning related to properties of individual stimuli was precluded.

### Auditory brightness of music and speech adapts on a trial-to-trial basis

**Figure 2:**
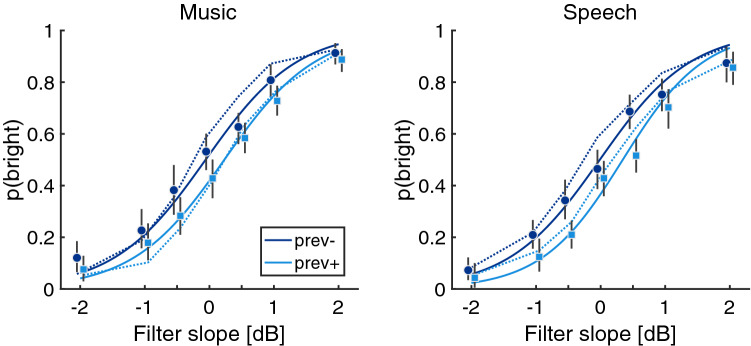
Experiment 1. Proportion of *bright* responses as a function of filter slope (presented in random order in the experiment) for music stimuli (left) and speech stimuli (right). For all visualizations, dark and light blue color corresponds to trials preceded by a trial with negative and positive filter slope, respectively. Circle and square symbols correspond to mean values per condition; solid lines correspond to fixed effects predictions of the GLME; dotted lines correspond to the predictions of a short-term decision-criterion model; black errorbars correspond to bootstrapped 95% confidence intervals.

In Exp. 1, we measured participants’ ability to judge the brightness of 2-s excerpts of popular music and speech utterances and their susceptibility to trial-by-trial adaptation of auditory brightness. We also assessed potential differences across the stimulus domains music and speech (presented in separate sessions). Figure 2 shows the proportion of *rather bright* responses from 19 participants (3 participants were removed because they did not fulfil the inclusion criterion, see Methods section). Apart from the empirical means, the figure shows two kinds of model predictions: the solid line corresponds to the fitted responses of a generalized linear mixed-effects (GLME) model (see supplementary material for detailed model statistics for all experiments), and the dashed line corresponds to the predictions of a signal-based model of the decision criterion (the Figures show the model with the smallest RMS error across the four experiments; see last subsection of Results).

Participants showed strong and consistent effects of filter slope ($\beta = 1.45$, 95% CI: [1.17, 1.74], p $<.001$), highlighting their general sensitivity to manipulations of auditory brightness. Clear indications of contrastive adaptation emerged, because brightness judgments were consistently affected by whether the filter slope of the previous trial was in the positive range (0.5, 1, 2 dB) or the negative range ($-2, -1, -0.5$ dB) ($\beta = -0.24$, [$-0.33, -0.16$], p $<.001$). As visible in Fig. 2, *rather bright* responses were more frequent for trials that were preceded by a negative filter slope (corresponding to a *dull* sound). Accordingly, participants judged sounds as relatively *dull*, when trials were preceded by a positive filter slope (corresponding to a *bright* sound). Note that an analysis that considered whether the slope of the previous trial was larger or smaller than the trial in relative terms yielded similar results ($\beta = -0.25, [-0.33, -0.16]$). There were no differences between music or speech stimuli in general ($\beta = -0.07$, [$-0.17, 0.03$], $p = .18$) and there was no strong interaction effect in the experiment ($|\beta | < 0.05$, $p > 0.09$). Because the stimulus domain did not appear to play any role for the effects of interest, only music stimuli were used in the subsequent experiments. Remarkably, the observed adaptation effect of auditory brightness already emerged on a trial-by-trial basis for excerpts of 2 s duration. In the subsequent experiment, we considered the hypothesis that the adaptation effect would increase in strength as a function of the adaptor duration.

### Adaptation effect partially increases with context duration

**Figure 3:**
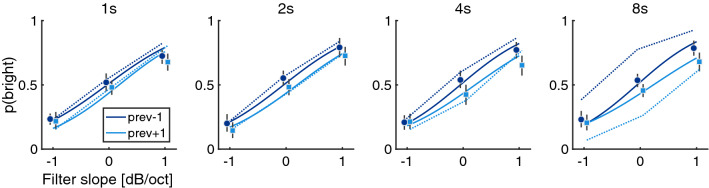
Experiment 2. Proportion of *bright* responses for contexts of different lengths (as indicated in the different panels) which preceded the target trials. The x-axis indexes filter slopes of target trials, dark or light line colors index filter slopes of previous adaptor trials (–1 or +1 dB/oct). Graphic conventions otherwise as in Fig. 2.

In Exp. 2, target and adaptor trials were interleaved with target durations of 2 s and adaptor durations of 1, 2, 4, or 8 s. As in all other experiments, participant were required to provide a response on every trial including the adaptors. Hence, participants did not know whether they were listening to test or adaptor trials and the purpose of the adaptor trials was not revealed to participants. Here, only target trials were analyzed. The filter slope of the adaptor trials (“context slope”) was $-1$ or 1 dB/oct, and the filter slope of the target trials (“target slope”) was $-1$, 0, or 1 dB/oct. Figure 3 shows the results from 18 participants that fulfilled the inclusion criterion (out of 22), separated in different panels according to the duration of the adaptor trials.

Responses were strongly affected by the target filter slope ($\beta = 1.34$, [1.07, 1.62], $p < .001$), and there was a relatively weak main effect of context filter slope ($\beta = -0.15$, [$-0.26, -0.03$], $p = 0.01$). The context length itself did not affect responses ($\beta = 0$, [$-0.05, 0.06$], $p =0.874$), and the hypothesized two-way interaction between context length and context filter slope was insignificant ($\beta = 0.01$, [$-0.06, 0.04$], p $=$ .694). Yet, there was a three-way interaction between context length, target filter slope, and context filter slope ($\beta = -0.08$, [$-0.15, -0.01$], p $=$ 0.017). This three-way interaction underlined that the context length only appeared to exert an effect for the non-negative target slopes, but for target slopes equalling −1 dB/oct, no adaptation was observed. Whether this interaction effect was an artifact of the specific experimental conditions (that contained only a small subset of the filter slopes from Exp. 1) or whether this pattern of result was related to other factors remains an open question. Nonetheless, the experiment suggested that for a majority of target slopes, an increase of the duration of preceding trials did have measurable effects on auditory brightness judgments. In the next experiment, we studied whether judgments would be affected by global shifts of the range of the presented filter slopes during entire experimental sessions.

### Long-term context strongly affects response behavior

**Figure 4:**
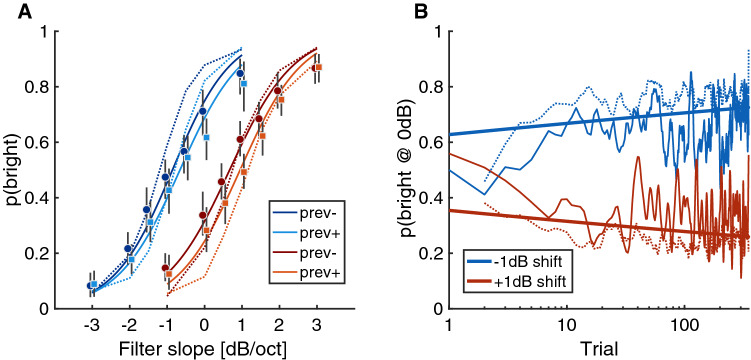
Experiment 3. **(A)** The blue and red graphs correspond to the global experimental contexts of presented filter slopes that were shifted by $-1$ and $+1$ dB, respectively. Whether previous trials had positive or negative filter slope is indexed by the different color shadings (see legend). **(B)** Depiction of the time-course of adaptation. Blue and red colors corresponds to experimental sessions with filter slopes from $-3$ to 1 dB/oct and $-1$ to 3 dB/oct, respectively. The thin lines correspond to the trajectory of *p(bright)* over a logarithmized trial index as assessed by averaging responses from all participants for the filter slope conditions $-1$, 0, and 1 dB/oct; the dotted lines correspond to the signal-based model (which does not have a history to compare with on the very first trial, hence no data is displayed there); the thick lines correspond to the fixed effects of the trial-wise GLME.

In Exp. 3, we used the same music excerpts and conditions of filter slopes as in Exp. 1, which were shifted 1 dB/oct down- or upwards in two different experimental sessions. Twenty participants fulfilled the inclusion criterion. As depicted in Fig. 4A, participants’ responses were highly susceptible to the induced global shift of the filter slopes, yielding a strong effect of global context ($\beta = -0.95$, [$-1.22, -0.68$], $p < .001$). For filter slopes of 0 dB/oct, the average proportion of *rather bright* responses equalled .66 for the downward shift of the global context and .31 for the upward shift, indicating a drastic contrastive context effect of 35 percentage points. As previously, we observed an effect of target slope ($\beta = 1.21$, [1.01, 1.40], $p <.001$), as well as a comparatively small effect of trial-to-trial adaptation ($\beta = -0.17, [-0.26, -0.07]$, $p = 0.001$), depending on whether the filter slope of the previous trial was above or below the mean slope of the experimental session. No interaction effects occurred.

To better characterize the time-course of response adaptation, we averaged responses from all participants per trial and context condition for those trials that contained one of the slope conditions $-1$, 0, and 1 dB/oct, which were the shared filter slopes from the two global contexts. These data were smoothed over time with a 10-point moving average filter. The resulting trajectory is displayed in Fig. 4B on a logarithmic time axis. The trajectories of the proportion of *bright* responses in the two global contexts start out similarly, but after around six to ten trials, clear differences start to emerge and stabilize. Besides the empirical averages, the figure also displays the GLME model (thick lines) run with the variables of filter slope, context, and an interaction term between a logarithmic trial index and context (with the same variables as random effects/slopes and intercepts plus random intercepts). Of specific interest for the current analysis was the interaction effect between context and trial index ($\beta = -0.05, [-0.08, -0.02]$, $p < .001$), visible in Fig. 4B as the divergent thick lines that represent the GLME model. Finally, the signal-based model takes a similar trajectory compared to the empirical data in the sense that it takes six to ten trials before differences between the two context conditions stabilize. In sum, the present results suggest that even though adaptation can be observed on a trial-by-trial basis, the full adaptation effect stabilizes after around eight trials of 2 s stimulus duration.

### Effects replicate for real-world acoustical manipulations

**Figure 5:**
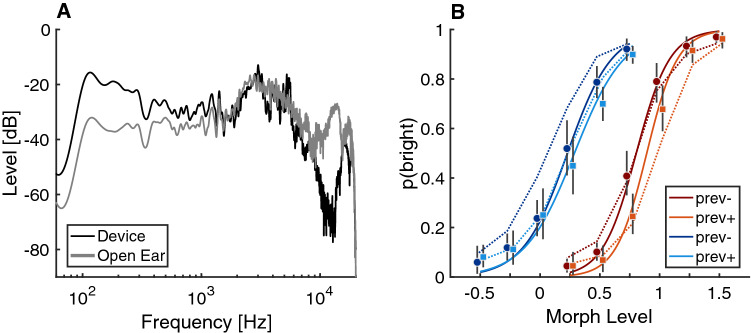
Experiment 4: **(A)** Smoothed frequency response of acoustical transfer functions for the hearable device in hear-through mode and the open ear. **(B)** Results from Exp. 4. Graphic conventions as in Fig. fig:exp3A.

In Exp. 4, we sought to replicate the effects observed in Exp. 3 with spectral manipulations from a real-world acoustical scenario. Instead of manipulating a linear spectral filter slope of the stimuli between 0.125 and 8 kHz, we used the acoustic transfer functions of a dummy head recorded in an acoustics lab^25^. In one condition, the dummy head had “open ears”, that is, it was not equipped with any hearing device. In the other condition, the dummy head wore commercially available earphones in a “hear through” mode that supposedly attempts to reproduce a pleasant acoustic transfer function. The magnitude response of these transfer functions is displayed in Fig. 5A, illustrating that the hearing device exhibited a response characterized by an amplification of low frequencies and attenuation of high frequencies compared to the open ear. We hypothesized that stimuli convolved with this transfer function (i.e., simulating the presentation in the respective acoustics lab) would be perceived as less bright compared to the open ear condition due to the reduced amount of gain in high frequencies. To use a comparable setup of conditions, we morphed between impulse responses by interpolating the transfer functions in the frequency domain (see “Methods”). In effect, a morph level of 0 would fully correspond to the Device condition, a morph level of 1 would fully correspond to the Open Ear condition, and a morph level of 0.5 would correspond to a half-way interpolation between the two. In order to achieve a broad range of experimental conditions, we extended the morph level to negative values up to $-0.5$, and positive values to 1.5, which allowed to overemphasize the acoustical differences between the two transfer functions in one or the other direction. Apart from the stimulus manipulation, the experiment was otherwise identical to Exp. 3.

Due to the Covid-19 pandemic, only 14 participants conducted the experiment, from which one participant needed to be removed due to a technical error. Results are shown in Fig. 5B and followed a similar pattern compared to Exp. 3. There were strong effects of morph level ($\beta = -6.27, [-7.35, -5.19]$, $p<.001$) and strong effects of context ($\beta = 1.33$, [1.09, 1.58], $p <.001$). There was also an effect of the previous trial, depending on whether the morph level of the previous trial were below or above the mean morph level of the respective session ($\beta = -0.32, [-0.49, -0.15]$, $p < .001$). There was a two-way interaction between the morph level and the context ($\beta = 1.18, [0.62, 1.75]$, $p < .001$) as well as a three-way way interaction between the context, the morph level, and the previous trial ($\beta = 0.3$, [0.03, 0.58], p $= .03$). These interaction effects correspond to the lack of differences between the two previous-trial conditions for negative morph levels, where stimuli tended to be perceived as *dull*, regardless of preceding context. Hence, results from Exp. 4 corroborate the notion that the adaptation of brightness perception is a process of relevance for hearing under real-world conditions.

### Signal-based model of decision criterion suggests short-term adaptation

**Figure 6:**
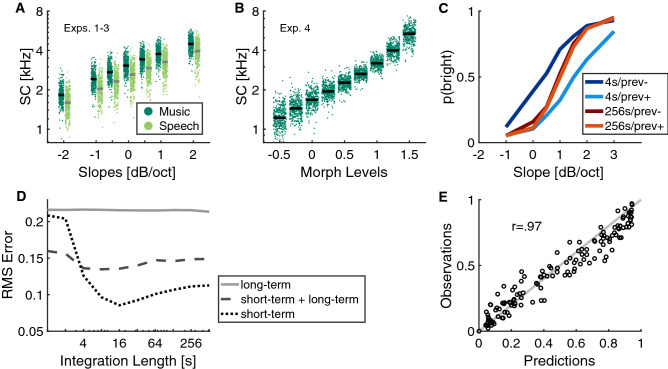
**(A)** Distribution of spectral centroid (SC) values for stimuli from different filter slopes from Exp. 1. Blue dots correspond to music, red dots correspond to speech, horizontal lines to the median value. **(B)** Distribution of SC values in Exp. 4. **(C)** Illustration of the effects of integration length for a short-term (ST) model for the example of Exp. 3. Dark and light colors correspond to trials preceded by trials with negative and positive filter slopes, respectively. Note that an integration length of 4 s overestimates trial-by-trial adaptation whereas an integration length of 256 s does not sufficiently reflect trial-by-trial adaptation. **(D)** RMS error of long-term, short-term + long-term, and short-term models for different integration lengths. **(E)** Scatterplot of predicted and observed data across all four experiments for the ST model with smallest RMS error (integration length: 16 s).

In order to further explicate the observed effects, we constructed simple models of task performance. We followed the literature that has used the spectral centroid (SC; center of gravity of the spectral energy distribution of the sound signal) as a feature for quantifying auditory brightness perception^3,26,27,36^. We focus on modelling an adaptive response criterion, which bears similarity to previous work on pitch content effects^19^. The distributions of SC values for the stimuli from Exp. 1 and Exp. 4 are shown in Fig. 6A,B (one SC value per stimulus). Speech utterances have slightly lower median SCs compared to music excerpts in Exp. 1, and the most extreme morph levels in Exp. 4 yield particularly low and high SCs.

Three different signal-based models were instantiated that differed in the type of decision criterion, to which SC values were compared on every trial. A first model was implemented by using a fixed long-term (LT) criterion, computed via the median SC value across all unprocessed stimuli for the respective stimulus domain (music or speech). This model would embody a decision criterion that would compare the brightness of a given trial with a long-term reference. A second model used a decision criterion computed via the median of SC values over a given integration length (which only considers the stimulus duration, but not silences in between). This model hence embodied a criterion that is based on information stored in auditory short-term (ST) memory. The decision criterion of a third model consisted of the mean criterion of the ST and LT models (ST+LT). Experiments were simulated for 50 virtual participants.

The model behavior is qualitatively illustrated in Fig. 6C, where extreme integration lengths of 4 and 256 s are displayed for the case of one context condition from Exp. 3: for very short integration lengths (4 s), the predicted trial-to-trial adaptation is clearly too large in comparison to the experimental data. But for very long integration lengths (256 s), the trial-to-trial adaptation is not accounted for any more (lines overlap).

Figure 6D shows the root-mean-square (RMS) prediction error pooled across all four different experiments as a function of integration length of the ST model. With RMS errors of more than 0.2, the LT model appeared to be incapable of explaining much of the experimental results. The combined ST+LT model yielded better performance, but the ST model was clearly most accurate, in particular for integration lengths beyond 8 s. The minimal RMS is reached for 16 s integration length and yields a model with Pearson correlation of r=.97 with the average empirical data across experiments (Fig. 6E). The predictions of this ST model with minimal RMS error are displayed in all results figures as dashed lines.

These modeling results are compatible with the findings from Exp. 3 in the sense that responses adapt within time spans of around a quarter of a minute. Hence, the adaptation effect appears to fall in the range of auditory short-term memory^28–31^.

## Discussion

We have reported a set of experiments that indicate consistent adaptation effects of auditory brightness judgments for naturalistic music and speech excerpts. Small but consistent effects emerged even after a single (hidden) adaptor trial. The strongest effect emerged between experimental sessions that presented an overlapping, yet different range of stimulus colorations. The measured effects were qualitatively similar for artificial spectral manipulations and manipulations based on transfer functions measured in a commercially available hearing device. We interpret these results as indicating an adaptation effect that grows with increasing duration of adaptor stimuli but levels off after around 8 trials of 2 s duration.

Several methodological differences between the present study and previous work from the literature on spectral contrast effects in musical instrument categorization^10–14^ deserve mention. First, these studies used sound source categorization tasks instead of the present judgements on an auditory attribute. Second, previous studies used isolated test tones at a fixed fundamental frequency instead of the present uncontrolled excerpts of popular music. Third, these studies used isolated sounds varying along a continuum between two sounds during full experimental sessions instead of the present design, which did not present a single music excerpt or speech utterance twice across a full experimental session. Nonetheless, we observed adaptation of brightness judgments on a trial-by-trial basis for naturalistic stimuli and even for listening with simulated earbuds in hear-through mode in our last experiment. That is, the present study extends the literature on spectral contrast effects by demonstrating consistent adaptive effects of auditory brightness under realistic acoustical conditions.

Our signal-based model of an adaptive decision criterion replicated the behavioral results across four experiments. Specifically, participants’ responses were modelled via a comparison of the spectral centroid of the current stimulus and the median spectral centroids from a prior context. Minimal error to the measured data was obtained for context length of 16 s and a correlation of r = .97. That is, despite its simplicity, the model accurately predicted the empirical data. These results may be interpreted as indicating that a decision criterion rooted in auditory short-term memory in principle suffices to account for the presented adaptation effects. This does not mean that we consider contributions of long-term memory to auditory brightness judgements as generally implausible. Our experiment only used unfamiliar stimuli; for familiar stimuli, such as familiar musical excerpts or human voices, it could be that the adaptation of brightness is more constrained.

It should be emphasized that the purpose of our model was primarily to shed light on the timescales of brightness adaptation as manifest in the present data. Effective models of auditory processing^32,33^ have proposed time constants for auditory adaptation that are at least one order of magnitude shorter compared to the present observations. For speech categorization, Feng et al.^15^ indicated that spectral contrast effects appear to be driven by fast-acting peripheral factors as well as arguably more slowly acting central processes. Yet, several other studies have suggested adaptive processes in auditory perception that extend over longer timespans. Modeling pitch discrimination, Raviv et al.^19^ found multi-trial dependencies with a similar, decision-criterion-based approach as in the present work. Chambers et al.^20^ even observed significant context effects in pitch-shift judgements across silent periods of 64 seconds. Studying sound texture perception, McWalter and McDermott^24^ suggested stimulus-dependent, multi-second integration windows to account for perceptual adaptation to texture statistics. One should note that the time constants found in these relatively slow-acting processes resemble time constants observed in the build-up of auditory stream segregation, which is known to stabilize after about 20 s^34^ (although the precise time point is stimulus dependent). That is, our results are in line with prior evidence for relatively slow-acting adaptive auditory processes, which appear to play a pertinent role in auditory brightness computations and more generally the formation of auditory attributes.

The attribute of auditory brightness has commonly been considered as a critical contributor to timbre perception and has been considered as being determined by the instantaneous spectrum^3,35,36^ processed in auditory cortex^37,38^. Yet, relatively little is known about neural underpinnings of spectral adaptation effects. Receptive fields in auditory cortex are rarely described to possess integration times longer than a few tenth of a second^39,40^. Stimulus specific adaptation^41^ has been suggested as a neural candidate mechanism for adaptation based on auditory sensory memory, spanning across different timescales beyond the range of seconds. On a behavioral level, we are not aware about prior reports on contrastive context effects in brightness perception, although there are some indirect hints. For instance, in a recent meta-analysis of timbre dissimilarity^42^, it has been suggested that perceptual timbre metrics are experiment specific. The present findings on the adaptivity of brightness perception, which plays an important role in general timbre dissimilarity^35^, provide a perspective from which reports of experiment-specificity may be reconsidered. In particular, the present results suggest that context-blind models^36,43^ of auditory brightness (and dissimilarity for that matter) are constrained, because important aspects of brightness perception appear to be fundamentally relative. By enhancing contrast, adaptive processes in auditory perception could serve as a way to facilitate information extraction from auditory scenes^44^. In the case of auditory brightness, adaptive processes pave the way for listeners to “forget” about potentially idiosyncratic coloration effects due to rooms, loudspeakers, or hearing devices and focus on local differences in a scene, much alike to visual color constancy^45^.

## Methods

### Participants

Participants were recruited online at the job board of the University of Oldenburg and received monetary compensation for their time. Exps. 1–3 each had 22 participants of self-reported normal hearing. In Exp. 1, participants had a mean age of 22.5 years (STD = 3.0, range: 19–28 years), and there were four participants with a background of on average 5.2 years of training on a musical instrument (range: 2–8 years). In Exp. 2, participants had a mean age of 22.6 years (STD = 2.2, range: 20–28 years), and there were 20 participants with on average 5.1 years of training on a musical instrument (range: 2–16 years). In Exp. 3, participants had a mean age of 23.1 years (STD = 2.7, range: 19–29 years), and there were four participants with on average 5.3 years of training on a musical instrument (range: 2–8 years). Due to the Covid-pandemic, there were only 14 participants in Exp. 4. These participants had a mean age of 23.8 years (STD = 2.9, range: 21–32 years), and there were ten participants with on average 8.2 years of training on a musical instrument (range: 2–16 years).

### Stimuli

Music excerpts were obtained from the so-called *Homburg dataset*^46^ that contains 10-s excerpts of popular music from different genres, available at an audio sampling frequency of 44.1 kHz. As stimuli, we selected excerpts from the musical genre categories *alternative*, *blues*, *folk/country*, *funk/soul/R’n’B*, and *pop* for their general similarity in long-term spectrum. The excerpts from the database were partitioned into 2-s portions that included a ramped-cosine fade-in and fade-out of 50 ms and were analyzed in terms of their spectral energy levels by computing the RMS energy of excerpts fed through a 5th-order Butterworth filterbank with center frequencies at 0.125, 0.25, 0.5, 1, 2, 4, 8, and 16 kHz. In order to use a stimulus set with relatively homogeneous frequency energy distribution, 369 candidate excerpts were chosen with mean RMS-deviations over bands of 1.8 dB (max 2.2 dB) relative to the grand mean over all of the 888 available excerpts, see Fig.1A. Music stimuli were drawn randomly without replacement on each trial such that participants encountered a specific musical excerpt only once per session.

As speech stimuli, five-word matrix sentences from three different languages were used^47^: Spanish, English, and German. For both Spanish and English sentences, there were five female and six male speakers; for German sentences, there were six female and five male speakers. The sentences had a mean durations of 2.0 s (STD = 0.26, range: 1.4–2.8 s) and signals were sampled at an audio sampling frequency of 44.1 kHz. Per speaker and language, the 10 excerpts that exhibited the smallest RMS deviation in terms of spectral energy compared to the gender-specific grand average were selected, see Fig.1B. This yielded 180 candidate sentences with mean RMS-deviations of 1.6 dB (max. 2.5 dB) for female speakers and 180 candidate sentences with mean RMS-deviations of 1.7 dB (max 2.8 dB) for male speakers compared to the gender-specific mean spectral distribution. Speech stimuli were presented in pseudo-randomized order such that no single speaker occurred on two subsequent trials.

In Exps. 1, the spectral slope of the stimuli was modified by feeding stimuli through the Butterworth filterbank mentioned above. The levels of individual channels was then adjusted before remixing as to yield different spectral slopes. Filter slopes were chosen in the range [$-2, -1, -.5, 0, +.5, +1, +2$] dB per octave in the frequency range between .125 and 8 kHz with a balance point of 1 kHz, and with no further alterations of filter weights below 0.125 kHz or above 8 kHz. See Fig.1C for the magnitude response of an exemplary music stimulus after different filter slopes were applied. Filter slopes were chosen randomly on every trial. Experiment 1 (and all other experiments) contained 50 trials per slope/morph level. In Exp. 2, the presentation of target trials (duration of 2 s) was interleaved with adaptor trials (durations of 1, 2, 4, or 8 s), but only target trials were analyzed. The filter slope of the adaptor trials (“context slope”) was chosen randomly as -1 or 1 dB/oct, and the filter slope of the target trials (“target slope”) was chosen randomly as $-1, 0$, or 1 dB/oct. In Exp. 3, the same music excerpts and conditions of filter slopes were used as in Exp. 1, but presented as part of two separate conditions (presented in separate sessions), in which filter slopes were shifted by 1 dB/oct down- or upwards.

In Exp. 4, stimuli were modified by means of artificial acoustic transfer functions that were generated by morphing between two measured acoustic transfer functions. Only the left channel of the measured transfer functions was used. One transfer function corresponded to a set of commercially available earphone in “hear-through” mode (called “Device” in the following). Another transfer function corresponded to the “Open-Ear” reference, see Fig. 5A for their magnitude responses. It is visible that the Device has a stronger low-pass characteristic compared to the Open Ear, and hence the latter was expected to sound brighter than the former. The morph between the two transfer functions was generated by linear inter- or extrapolation in the spectral log-magnitude domain and the unwrapped phase domain. A morph level of 0 thus corresponds to the Device, whereas a morph level of 1 corresponds to the Open Ear. Accordingly, negative morph levels correspond to exaggerating the properties of the Device relative to the Open Ear and positive levels to exaggerating the properties of the Open Ear relative to the Device.

### Apparatus

In Exps 1–3, listeners were tested individually in a sound-proof booth and provided responses on a computer keyboard. Sounds were presented with an RME FIREFACE audio interface at an audio sampling frequency of 44.1 kHz and 24 bit resolution. They were presented diotically over Sennheiser HDA 650 headphones at 70 dBA sound pressure level, as calibrated by a Norsonic Nor140 sound-level meter with a G.R.A.S. IEC 60711 artificial ear to which the headphones were coupled. Headphones were equalized to obtain a flat magnitude response at the KEMAR eardrum by using regularized inversion as implemeted in the AKtools toolbox^48^. Transfer functions were measured using a GRAS 45BB-12 KEMAR Head & Torso with anthropometric pinnae in an acoustics lab with reverberation time $T_{60}$ of around 0.45 s with loudspeakers in a frontal direction, see Schepker et al.^25^ for details. Due to the Covid-19 pandemic, in Exp. 4 listeners were not tested in sound-proof lab, but in a quiet space. The same headphones were used in conjunction with an RME BabyFace audio interface using the same presentation level as in the previous experiments.

### Procedure

All experiments were administered in two sessions on separate days. In Exp. 1, music stimuli were presented in one session and speech stimuli in another. In Exp. 2, the same experimental conditions were presented in two distinct sessions. In Exps. 3 and 4, the global context factor was split across sessions with one context per session. Experiments 1 and 3 contained 350 trials per experimental session (700 overall) and experiments 2 and 4 contained 300 trials per experimental session (600 overall). The order of presentation of the global context conditions was counterbalanced across participants. After the completion of the first session, participants filled out a biographical questionnaire.

Every trial consisted of the presentation of a stimulus followed by a prompt inquiring whether the sound of the excerpt was rather bright or rather dull (*“War der Klang eher zu hell oder zu dunkel?”*). During the course of an experiment, none of the music or speech excerpts was presented twice to participants. In the experimental instructions, participants were asked to imagine listening to an entire piece of music or a conversation with the presented sound settings and judge whether the setting would be perceived as rather too bright or too dull for this purpose. To provide a general sense of orientation, participants listened to 6 example trials before the start of the main experiments, for which “correct” responses were indicated by denoting trials with negative filter slopes (or morph levels below 0.5 in Exp. 4) as dull and positive slopes (morph levels above 0.5 in Exp. 4) as bright. All participants provided informed consent. The experimental procedure was in accordance with the Declaration of Helsinki and was approved by the ethics committee of the University of Oldenburg (EK/2019/092).

### Data analysis

By visual inspection of the data, it was noticed that not all participants yielded interpretable results in Exps. 1–3. For that reason, it was decided not to include participants in the analysis if they showed smaller gains than 10 percentage points in proportion bright scores per 1 dB/oct increase of spectral slope, which was assessed by comparing the two most extreme slopes per session. That is, in Exps. 1 and 3, participants were required to show a gain of 40 percentage points from $-2$ dB/oct to +2 dB/oct slopes in order to be included in the analysis, and a gain of 20 percentage points from $-1$ dB/oct to +1 dB/oct in Exp. 2. We did not observe any relationship between musical training and whether or not participants needed to be removed from the analysis.

Trial-level accuracy was analyzed using a generalized linear mixed-effect (GLME) model^49^ as implemented in the *fitglme* class in MATLAB (https://www.mathworks.com), using a logit link function and a binomial distribution of the response variable. Our models comprised by-participant random slopes and intercepts for all fixed effects factors as well as by-item random intercepts. All binary categorical predictors were effects-coded. All variables with more than two levels were numerically coded without normalizing predictors. The full analysis results are provided in the Supplementary Materials. The fitted statistical models are plotted in all results figures (Figs. 2, fig:exp2, fig:exp3, fig:exp4) as solid lines. Square brackets in the text indicate 95% confidence (i.e., compatibility) intervals for a given estimate.

### Modeling the decision criterion

A context-dependent model of the decision criterion was constructed to further explicate the timescales of brightness adaptation. The model was evaluated by simulating the same experiments that human participants conducted with 50 simulations (i.e., virtual participants) per experiment. The averaged results for the model with minimal error are plotted as dotted lines in the results figures. The model was based on the spectral centroid (SC), which corresponds to the center of gravity of the spectral energy distribution and has been shown to reliably correlate with brightness ratings of isolated musical instrument sounds^3,26,27^. SCs were computed via the magnitudes of a Fast Fourier Transform (FFT) of the signal; all magnitudes below the threshold of -90 dB full scale were set to zero^50^. The resulting magnitudes were grouped by using an equivalent-rectangular-bandwidth (ERB) filterbank^51^ with 128 bands. The formular for the SC reads as, $SC = \frac{\sum _j f_j \cdot E_j}{\sum _j E_j}$, where $f_j$ denotes the center frequency and $E_j$ the magnitude of the j-th ERB-band. Fig. 6A and B show the distribution of SC values for all three stimulus types (filtered music and speech from Exp. 1–3, and filtered music from Exp. 4).

The model formulation was implemented as a weighted sum of a short-term and long-term criterion. The former embodied an adaptive internal reference of listeners and the latter embodied a potentially fixed internal reference. Because the distribution of SC values of the excerpts was skewed, using the median was preferable compared to an implementation using a leaky-integrator that has frequently been used for auditory short-term memory^52,53^. The decision criterion is given by

1 $$\begin{aligned} SC[n] > \alpha \cdot med\left( \left\{ SC[n-1-k], ..., SC[n-1]\right\} \right) + (1-\alpha ) \cdot med\left( \left\{ SC[1], ..., SC[N] \right\} \right) \end{aligned}$$

where *k* indexes the trial at time *L* seconds before trial *n*, and *N* denotes the total number of trials per experimental session. That is, in every trial the model compared the current SC with the median of the SCs of the excerpts in the last *L* seconds (*L* denoting the integration length). Silences between or within trials were not considered. Hence, with $\alpha = 1$ this model collapses to the short-term model described above. With $\alpha = 0.5$, the model corresponds to a weighting of a short- and long-term criterion. With $\alpha = 0$, the model is solely based on a fixed long-term criterion. To account for internal noise and hence response lapses independent of the experimental condition, the model included a lapse rate of 5% of trials, in which case the model would provide a response other than indicated by the criterion in Eq. (1).

## Supplementary Information

Supplementary Information.

## Data Availability

The datasets analyzed during the current study as well as selected sound examples are available at https://github.com/Music-Perception-and-Processing/adaptive-auditory-brightness.
