# Supplementary material for: Adaptive auditory brightness perception
Source: Sci Rep. 2021 Nov 2;11:21456. doi: 10.1038/s41598-021-00707-7 (PMC8563846; doi:10.1038/s41598-021-00707-7)
Supplement: Supplementary file 1 — Supplementary Information. [file 41598_2021_707_MOESM1_ESM.pdf]

Supplementary materials (Adaptive auditory brightness perception)

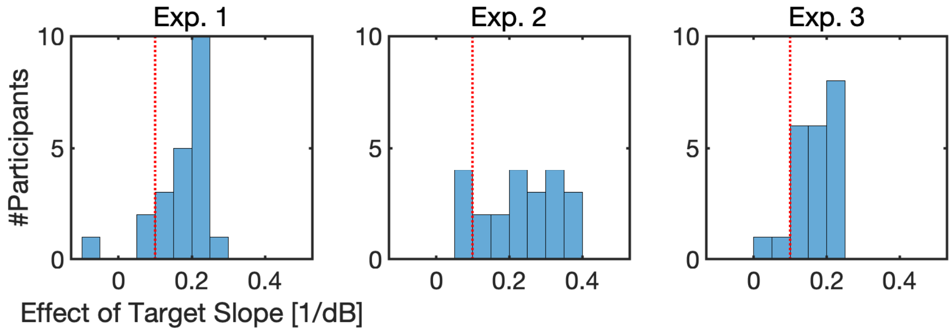

Figure 1. Outlier participants in Exps. 1–3. Plots show the effect of the spectral slopes across participants as assessed by subtracting p(bright) values for the minimum spectral slope condition from p(bright) values for the maximum spectral slope condition and dividing by the spectral slope range. Participants with effects less than 0.1/dB were removed from the analysis in all three experiments (in Exps. 1 and 3, this corresponds to a gain of 40 percentage points from -2 dB to +2 dB slopes, and in Exp. 2, this corresponds to a gain of 20 percentage points from the -1 dB to +1 dB spectral slope condition). This criterion resulted in the removal of three participants in Exp. 1, four participants in Exp. 2, and two participants in Exp. 3. In Exp. 4, one participant needed to be removed due to a technical error.

Table 1. Exp. 1. Results from the GLME model with the independent variables domain (music vs. speech), slope (-2, ..., +2), previous trial bright, and interaction terms between factors (Iindexed by ":"). Model statistics: AIC = 65715, $R^2 = .78$.

Variable	β	CI low	CI high	t-value	p-value
intercept	-0.19	-0.43	0.05	-1.56	0.119
domain	-0.07	-0.17	0.03	-1.32	0.186
slope	1.45	1.17	1.74	10.03	$< .001$
prev	-0.24	-0.33	-0.16	-5.75	$< .001$
domain:slope	0.05	-0.02	0.11	1.36	0.175
domain:prev	-0.04	-0.1	0.02	-1.27	0.203
slope:prev	0.05	-0.01	0.10	1.66	0.097
domain:slope:prev	0.04	-0.01	0.10	1.44	0.149

Table 2. Exp. 2. Results from the GLME model with the independent variables context length ("c-length": 1, 2, 4, 8 sec), target slope (-1, 0, +1), context slope ("c-slope": -1, +1), and interaction terms between factors (lindexed by ":"). Model statistics: AIC = 40324, $R^2 = .49$.

Variable	β	CI low	CI high	t-value	p-value
intercept	-0.1	-0.38	0.17	-0.74	0.461
c-length	0.00	-0.05	0.06	0.16	0.874
slope	1.34	1.07	1.62	9.49	$< .001$
c-slope	-0.15	-0.26	-0.03	-2.56	0.01
c-length:slope	0.00	-0.06	0.07	0.08	0.936
c-length:c-slope	-0.01	-0.06	0.04	-0.39	0.694
slope:c-slope	0.05	-0.08	0.19	0.77	0.443
c-length:slope:c-slope	-0.08	-0.15	-0.01	-2.39	0.017

Table 3. Exp. 3. Results from the GLME model with the independent variables context (-1, +1 dB shift), slope (-2, ..., +2), previous slope (negative, positive), and interaction terms between factors (lindexed by ":"). Model statistics: AIC = 68318, $R^2 = .67$.

Variable	β	CI low	CI high	t-value	p-value
intercept	-0.01	-0.3	0.29	-0.04	0.968
context	-0.95	-1.22	-0.68	-6.86	$< .001$
slope	1.21	1.01	1.40	12.16	$< .001$
prev	-0.17	-0.26	-0.07	-3.46	0.001
context:slope	-0.03	-0.09	0.03	-0.91	0.361
context:prev	-0.01	-0.08	0.05	-0.44	0.658
slope:prev	-0.01	-0.05	0.04	-0.37	0.711
context:slope:prev	0.03	-0.02	0.07	1.25	0.212

Table 4. Exp. 3. GLME results for trial-wise analysis with the independent variables context (-1, +1 dB shift), slope (-2, ..., +2), and the interaction terms between the context and the logarithmic trial number (context:log-trial). Model statistics: AIC = 68237, $R^2 = .67$.

Variable	β	CI low	CI high	t-value	p-value
intercept	-0.04	-0.32	0.24	-0.28	0.78
context	-0.56	-0.89	-0.23	-3.32	0.001
slope	1.2	1.01	1.39	12.2	$p < .001$
context: log-trial	-0.05	-0.08	-0.02	-3.49	p <.001

Table 5. Exp. 4. GLME results for trial-wise analysis with the independent variables context (-1, +1 dB shift), morph (-0.5, ..., 1.5), the slope of the previous trial (negative, positive), and interaction terms (indexed by ":"). Model statistics: AIC = 44473, $R^2 = .96$.

Variable	β	CI low	CI high	t-value	p-value
intercept	2.45	1.95	2.95	9.67	$< .001$
context	1.33	1.09	1.58	10.67	$< .001$
morph	-6.27	-7.35	-5.19	-11.4	$< .001$
prev	-0.32	-0.49	-0.15	-3.70	$< .001$
context:morph	1.18	0.62	1.75	4.13	$< .001$
context:prev	-0.04	-0.19	0.11	-0.57	0.568
morph:prev	-0.01	-0.28	0.26	-0.07	0.941
context:morph:prev	0.30	0.03	0.58	2.16	0.03
